# The application progress of artificial intelligence in osteoporosis diagnosis

**DOI:** 10.3389/frai.2025.1699762

**Published:** 2025-11-19

**Authors:** Wenwei Zhang, Dongxu Tai, Siwen Kang, Keda Li

**Affiliations:** 1 Liaoning University of Traditional Chinese Medicine, Shenyang, China; 2 Liaoning University of Traditional Chinese Medicine Affiliated Hospital, Shenyang, China

**Keywords:** osteoporosis, artificial intelligence, machine learning, fracture risk, diagnosis

## Abstract

Osteoporosis (OP) is a systemic bone metabolic disorder characterized by a decrease in bone mineral density (BMD) and damage to the trabecular bone microarchitecture. With the increasing global aging population, the incidence of OP has been rising annually, particularly among elderly women, making it a significant public health issue. Traditional diagnostic methods such as dual-energy X-ray absorptiometry (DXA), quantitative computed tomography (QCT), and magnetic resonance imaging (MRI) are effective, but they also have certain limitations. Artificial intelligence (AI) technology is playing an increasingly important role in the management of osteoporosis. Through machine learning (ML), image processing, and data analysis, AI can accurately assess bone density, fracture risk, and other factors, improving the early diagnosis rate of OP and providing strong decision support for clinicians to optimize treatment plans and enhance treatment outcomes. However, it also faces challenges such as AI model interpretability, insufficient diversity in training data, lack of clinical validation, and issues related to privacy protection and ethics. Addressing these problems is crucial for promoting the widespread application of AI technology in this field. As technology continues to advance, AI will become an indispensable part of OP research and clinical applications, driving the development of personalized treatment and precision medicine.

## 1 Introduction

OP is a systemic bone metabolic disorder characterized by a reduction in BMD and trabecular bone microarchitecture damage (Zhang et al., 2025). This condition typically arises when the rate of bone resorption exceeds that of bone formation, leading to a loss of bone mass, which results in decreased bone strength and an increased risk of fractures (Anam and Insogna, 2021). With the global aging population, the incidence of OP has been increasing annually, becoming a significant public health challenge, particularly among elderly women (Zhang et al., 2024). OP not only severely impacts the quality of life of patients but also places a significant burden on the healthcare system, leading to high medical costs and socioeconomic expenses (Vendrami et al., 2023). OP often presents no obvious symptoms in its early stages, and many patients fail to receive timely diagnosis and treatment before fractures occur, leading to serious health consequences (Surlari et al., 2023). Therefore, early diagnosis and effective treatment are particularly important. The traditional diagnostic methods for OP mainly include DXA, QCT, and MRI (Tse et al., 2021). Although these methods are effective in assessing bone density and identifying changes associated with osteoporosis, they also have certain limitations (Laskey, 1996). Although QCT can provide more precise three-dimensional bone density images, it results in higher radiation exposure compared to DXA (Njeh et al., 1999). Although MRI has a high sensitivity in detecting bone marrow changes, it is costly and has limited accessibility for routine OP screening, which poses certain practical limitations (Bloem et al., 2025). These limitations highlight the urgent need to develop OP early detection tools that are more accessible, accurate, and low-risk.

AI is a key field in computer science, aimed at enabling computer systems to simulate human cognitive processes and intelligent behaviors, granting them human-like thinking abilities (Li et al., 2023). The primary goal is to enhance the system’s intelligence by processing and analyzing multidimensional data, thereby constructing intelligent models capable of prediction and decision-making (Xu et al., 2021). By leveraging the theories from disciplines such as mathematics, logic, computer science, and biology, AI has achieved groundbreaking innovations in the medical field. These technological advancements not only enhance the capabilities of physicians in performing medical tasks but also assist medical technicians in solving many time-consuming and labor-intensive challenges that were previously difficult to address. With the continuous development of ML and deep learning (DL), AI systems are now able to efficiently analyze vast amounts of medical data, including patient records and imaging data, providing profound insights for diagnosis, treatment planning, and patient care (Rana and Bhushan, 2022). In addition, AI-driven medical tools and systems, such as decision support and diagnostic algorithms, assist healthcare professionals in making more accurate and timely clinical decisions (Magrabi et al., 2019). This collaboration not only optimizes healthcare processes but also significantly enhances patient treatment outcomes, holding immense potential to disrupt medical practice, research, and education. It ushers in a new era for precision medicine and data-driven healthcare innovation. In the management of osteoporosis, the application of AI technologies is playing an increasingly important role. Through ML algorithms, imaging processing techniques, and data analysis, AI is capable of accurately assessing bone density, fracture risk, and other factors. These technologies not only improve the early diagnosis rate of OP but also provide strong decision support for clinicians, aiding in the optimization of treatment pathways and enhancing treatment outcomes (Smets et al., 2021). With the continuous advancement of technology, AI is gaining increasing attention in the research and clinical application of osteoporosis, becoming an indispensable part of future OP management. This article aims to review the progress of AI in the diagnosis and risk prediction of osteoporosis. It first reviews the research on AI in OP risk prediction, then explores the application of AI in combination with traditional imaging diagnostic technologies such as X-ray, DXA, CT, QCT, and MRI. Finally, it analyzes the potential, future development directions, and challenges of AI in clinical applications for osteoporosis, in order to provide references for clinical diagnosis, treatment, and research in OP.

## 2 The application of AI in OP risk prediction

Accurate prediction of osteoporotic fracture risk is crucial for early intervention and reducing fracture incidence. With the continuous advancement of AI technology, many studies have begun to utilize AI models for fracture risk prediction (Table 1). Compared to traditional prediction methods, AI-based OP risk prediction can analyze multi-dimensional data, improving the accuracy of risk assessment. This significantly enhances prediction precision, providing a more reliable basis for early clinical intervention. In a study involving 1,224 men and women, Fasihi L et al. employed various ML algorithms, such as random forests (RF) and support vector machines (SVM), to predict the risk of osteoporosis. The results showed that certain algorithms outperformed traditional methods, particularly in female patients, where the AUROC value reached 0.95, demonstrating extremely high predictive accuracy (Fasihi et al., 2022). Ulivieri FM et al. evaluated an AI-based model for predicting fragility fractures, the Bone Strain Index (BSI), by analyzing spinal X-ray and DXA data from 172 female patients using artificial neural networks (ANNs). The study selected five variables: age, age at menopause, BMI, femoral total bone mineral content (FTot BMC), and femoral total bone strain index (FTot BSI). The results demonstrated that the ANN model achieved an accuracy of 79.36%, a sensitivity of 75%, and a specificity of 83.72% (Ulivieri et al., 2021). These studies provide strong support for the prediction of OP and its associated fracture risks, especially demonstrating great potential for application in female patients.

**Table 1:** Application of AI in OP prediction.

Researchers	Sample Size	AI Model	Performance indicators
Fasihi et al. (2022)	1,224 patients	RF and SVM	The prediction accuracy is higher in the female population, with an AUROC value of 0.95.
Ulivieri et al. (2021)	172 female patients	ANNs	The ANN achieved a prediction accuracy of 79.36%, with a sensitivity of 75% and specificity of 83.72%.
Kong et al. (2020)	2,227 participants (with 1,257 females)	CatBoost, SVM, and logistic regression models	The CatBoost model outperforms FRAX, with an AUC value of 0.688, demonstrating higher accuracy compared to traditional models.
Zhang et al. (2024)	80 patients	SVR model	The optimized SVR model (MSE ≤ 0.014, R2 ≥ 0.93) showed high agreement with QCT/FEA predictions.
Bodden et al. (2023)	420 patients	vBMD model	The vBMD model outperforms the fracture status and count models (AIC = 165.2).

Kong et al. (2020) developed a ML-based fracture risk prediction model using CatBoost, SVM, and logistic regression models, and validated it in the prospective Ansung cohort. The research results show that the CatBoost model performs the best in predicting hip fragility fractures, with an AUC value of 0.688, significantly outperforming both the FRAX tool (0.663) and traditional models. Zhang et al. (2023) developed a ML model based on QCT images to predict proximal femoral strength. Femoral strength was calculated using finite element analysis (QCT/FEA), and 50 predictive variables were extracted. The SVR model, optimized through feature selection and dimensionality reduction, performed the best (MSE ≤ 0.014, R2 ≥ 0.93). The prediction results were in high agreement with QCT/FEA, demonstrating the potential of this model in clinical assessments. Bodden et al. (2023) predicted vertebral fragility fractures (VF) by using a convolutional neural network (CNN) framework to automatically extract volumetric BMD (vBMD) from routine CT images. The study included 420 patients and analyzed the relationship between vBMD and fractures. The results indicated that patients with low vBMD had a higher risk of fractures across all segments of the spine, particularly in the L1-5 region. Compared to traditional prediction models based on fracture status and count, the vBMD model performed better (Akaike’s information criteria, AIC = 165.2). These studies also demonstrated the accuracy and application potential of AI in predicting OP fractures, particularly in personalized prediction through the extraction of features such as bone mineral density from imaging data. In conclusion, compared to traditional prediction methods, AI models can handle more dimensions of data and provide more precise risk assessments. In the future, with further development and optimization of technology, the application of AI in OP risk prediction is expected to provide more reliable evidence for early clinical intervention, helping to reduce fracture incidence and improve patients’ quality of life.

## 3 The application of AI in OP diagnosis

Many studies have shown that the integration of artificial intelligence with X-ray, DXA, CT, QCT, and MRI provides more precise and intelligent solutions for the diagnosis of OP. AI can automatically extract key features from images, improve diagnostic efficiency, reduce errors, and assist in early risk identification, thereby enabling personalized treatment plans. For details, refer to Table 2.

**Table 2:** The application effectiveness of AI across different image modalities.

Image modalities	Researchers	Key technical approaches	Performance indicators
X-ray and DXA	Areeckal et al. (2018)	Cortical radiomorphometry and trabecular texture analysis andneural network classifier	Bone Defect Area Segmentation Accuracy: 89.9, 93.5%. Classifier Training Accuracy: 94.3%, Testing Accuracy: 88.5%.
	Wani and Arora (2023)	CNN-based Transfer Learning (AlexNet, VggNet-16/19, ResNet)	Pre-trained AlexNet Model Accuracy: 91.1%.
	Yang et al. (2021)	DL-based Resblock Model	Ulna Dice Coefficient: 0.9835, Jaccard Index: 0.9680 Radius Dice Coefficient: 0.9874, Jaccard Index: 0.9751.
	Mao et al. (2022)	CNN-based Classification Model (Combining Anteroposterior and Lateral X-ray Views)	Best Model AUC = 0.909–0.937.
	Lin et al. (2024)	AI Model + Chest X-ray Screening for High-Risk OP Patients to Guide DXA Examination	Screening group DXA examination rate: 12.8%, new OP diagnosis rate: 75.2% Screening group OP detection rate: 11.1% (control group: 1.1%), OR = 11.2 (*P* < 0.001).
CT and QCT	Yang et al. (2022)	AI measurement of the HU of the thoracic spine and first lumbar vertebra in routine chest CT	Thoracic spine combined diagnosis AUC: 0.831, single vertebra AUC: 0.972. For every increase of 10 HU, bone mass decreases / OP risk decreases by 32–44% / 61–80%, respectively.
	Chen et al. (2023)	ML segmentation and radiomic texture analysis	Bone density abnormalities / OP prediction overall accuracy: 0.90 ± 0.05.
	Yasaka et al. (2020)	A DL model based on unenhanced abdominal CT images.	CNN predicted BMD and DXA measured BMD Pearson correlation coefficient: 0.852 (internal), 0.840 (external). AUC for diagnosing OP: 0.965 (internal), 0.970 (external).
	Fang et al. (2021)	DCNN: U-Net + DenseNet-121 (BMD calculation)	Average Dice coefficient for automatic and manual lumbar vertebra segmentation: 0.782–0.823. Correlation coefficient (r) between automatic BMD calculation and QCT measured BMD: r > 0.98.
MRI	Deniz et al. (2018)	An automatic proximal femur segmentation method based on DCNN	Segmentation precision and recall are both 0.95.
	Yabu et al. (2021)	CNN-based fusion model (VGG16, VGG19, DenseNet201, ResNet50)	Model AUC: 0.949. Sensitivity: 88.1%, Specificity: 87.9%, Accuracy: 88.0%.

### 3.1 AI in combination with X-ray and DXA

In the diagnosis of osteoporosis, X-ray and DXA are commonly used diagnostic methods. X-ray helps detect fractures or bone abnormalities by evaluating changes in bone density, but its sensitivity and specificity have limitations in early diagnosis. In contrast, DXA is considered the gold standard for OP diagnosis (Morgan and Prater, 2017). DXA can accurately measure BMD and, by comparing it to standard values, determine whether a patient is at risk for OP or osteopenia. However, the availability of DXA equipment is limited, and in many countries (Kanis et al., 2021), there is a significant shortage of DXA machines. The emergence of AI technology provides a new solution for the diagnosis of OP. By combining AI with X-ray and DXA, AI can automatically analyze images, extract key bone density features, and detect subtle changes, improving the accuracy and speed of diagnosis. AI can also integrate with other health data to provide more comprehensive diagnoses and personalized treatment recommendations. Especially in resource-limited areas, it can supplement the shortage of DXA equipment and expand the coverage of screening.

Areeckal et al. (2018) proposed a low-cost automated tool for the early diagnosis of OP using wrist and hand X-ray images. The tool combines cortical radiomorphometry and trabecular texture analysis, utilizing a neural network classifier to distinguish between healthy and low bone mass subjects. Experimental results showed that the segmentation method achieved accuracy rates of 89.9 and 93.5% in detecting bone loss regions. The classifier achieved training and testing accuracy rates of 94.3 and 88.5%, respectively. This technology provides an effective tool for low-cost, large-scale screening of OP risk. It is especially suitable for developing countries or regions with limited equipment and resources. Wani and Arora (2023) proposed a CNN-based method for detecting OP using X-ray images. The study used a dataset of 381 knee X-ray images and employed transfer learning techniques with pre-trained models like AlexNet, VggNet-16, ResNet, and VggNet-19 for classification. The results showed that the pre-trained AlexNet model achieved an accuracy of 91.1%, significantly higher than the 79% accuracy when no pre-trained network was used. Yang et al. (2021) proposed a DL-based residual block (Resblock) model for the automatic segmentation of the ulna and radius in dual-energy X-ray images. The study used data from 360 subjects and evaluated the model performance through five-fold cross-validation. The results showed that the proposed model outperformed traditional methods in segmentation accuracy, with average Dice coefficients of 0.9835 and 0.9874 for the ulna and radius, respectively, and Jaccard indices of 0.9680 and 0.9751. Mao et al. (2022) proposed a screening method based on CNNs for detecting OP and osteopenia through lumbar spine X-rays. The study collected data from 6,908 participants, using DXA-measured BMD values as the reference standard. Three types of CNN models were developed for classification, and the impact of including clinical covariates (such as age, gender, and BMI) on diagnostic performance was explored. The results showed that the model based on antero-posterior and lateral X-rays performed best in diagnosing osteoporosis, with an AUC ranging from 0.909 to 0.937. Lin et al. (2024) conducted a randomized controlled trial to evaluate the effectiveness of an AI model in identifying high-risk OP patients through chest X-rays and performing DXA screening. The study included 40,658 participants, of whom 12.1% were identified as high risk. In the screening group, 12.8% underwent DXA, and 75.2% were diagnosed with new osteoporosis. The OP detection rate in the screening group was significantly higher than in the control group (11.1% vs. 1.1%), with an odds ratio of 11.2 (*p* < 0.001). The study highlights the clinical value of combining chest X-ray imaging with AI technology in the early diagnosis of OP. AI technology automates the analysis of X-ray images, helping to detect osteoporosis more quickly and accurately, thereby reducing diagnosis time and improving screening efficiency. This method holds great potential, especially in resource-limited areas.

In conclusion, the combination of AI with X-ray and DXA provides a more intelligent and precise solution for the diagnosis of OP, significantly improving diagnostic accuracy. Through DL, AI can precisely segment and classify OP regions, enhancing early detection accuracy. However, AI still relies on high-quality equipment and diverse datasets, and the limitations of current datasets may affect diagnostic outcomes across different populations. Additionally, challenges remain in data acquisition, model interpretability, and infrastructure development. As AI technology continues to advance, it is expected to play an increasingly important role in OP diagnosis, improving patient care and treatment outcomes.

### 3.2 The combination of AI with CT and QCT

CT and QCT also play important roles in the diagnosis of osteoporosis. CT helps detect fractures and changes in bone structure through high-resolution scans, but due to its higher radiation dose, it is generally not used as a routine screening tool (Liebl et al., 2013; Huda et al., 2002). QCT, on the other hand, can accurately quantify bone density, making it particularly suitable for assessing areas like the spine and femur (Chiba et al., 2022). Compared to traditional DXA, QCT offers higher precision and spatial resolution, distinguishing between cortical bone and trabecular bone density, thereby providing a more comprehensive analysis of bone quality (Brunnquell et al., 2021). However, the high radiation dose and cost of CT and QCT remain their limitations. Combining CT and QCT with AI can significantly enhance the sensitivity, specificity, and accuracy of OP diagnostic tools, addressing some of the shortcomings of traditional methods. Yang et al. (2022) explored the use of AI to measure the attenuation values (HU) of the thoracic and first lumbar vertebrae in routine chest CT images for OP screening. The study found that with age, especially in postmenopausal women, the CT attenuation values of the thoracic and first lumbar vertebrae decreased, and these values were significantly correlated with BMD. The attenuation values demonstrated high predictive and diagnostic efficacy. For every 10 HU increase in CT values, the risk of osteopenia or OP decreased by 32–44% and 61–80%, respectively. The combined diagnostic efficacy of all thoracic vertebrae was higher than that of a single vertebra, with AUC values of 0.831 and 0.972, respectively. This study suggests that AI-assisted chest CT can effectively screen high-risk populations and reduce the incidence of fractures. Chen et al.’s study developed a bone density screening tool combining ML segmentation and radiomic texture analysis, using chest low-dose CT (LDCT) for bone density prediction. By analyzing 197 patients, the study demonstrated the high accuracy of the automatic segmentation model and two-level classifier in detecting abnormal bone density and osteoporosis, with an overall prediction accuracy of 0.90 ± 0.05 (Chen et al., 2023). A study investigated the prediction of lumbar vertebral BMD using a DL model based on unenhanced abdominal CT images. The results showed that the BMD predicted by the CNN model was highly correlated with the BMD measured by DXA, with Pearson correlation coefficients of 0.852 and 0.840, respectively. The model demonstrated excellent performance in diagnosing osteoporosis, with AUCs of 0.965 and 0.970 for the internal and external validation datasets, respectively (Yasaka et al., 2020). This study shows that the CNN model in abdominal CT images can accurately reflect the BMD measured by DXA, providing a new approach for non-invasive BMD assessment.

Fang et al. developed an automated method based on deep CNN (DCNN) for vertebral segmentation and BMD calculation in CT images. The study used data from 1,449 patients, with U-Net for vertebral segmentation and DenseNet-121 for BMD calculation. The results showed good correlation between automated and manual segmentation, with average Dice coefficients for lumbar vertebrae of 0.823, 0.786, and 0.782. Additionally, the BMD calculated by the automated method showed high correlation with the BMD measured by QCT (r > 0.98). This method enables the automatic identification of osteoporosis, osteopenia, and normal bone mineral density, providing support for clinical OP screening (Fang et al., 2021). In conclusion, AI combined with CT and QCT offers significant advantages in the screening and diagnosis of osteoporosis, such as improved diagnostic accuracy, automated segmentation and classification, and more precise bone quality analysis. However, the high radiation dose and equipment costs of CT and QCT limit their widespread adoption. Additionally, the AI models suffer from insufficient diversity in their training datasets, and clinical validation is still limited, affecting their application across different populations. Therefore, despite the great potential of AI in imaging diagnostics, issues related to data diversity and interpretability must be addressed in order to achieve broader clinical applications.

### 3.3 The combination of AI and MRI

MRI has high tissue contrast and is commonly used for soft tissue imaging (Bruno et al., 2019). Compared to X-ray and CT, MRI is more sensitive in detecting early changes in bone marrow, which helps in predicting fracture risk (Zhang et al., 2021). When combined with artificial intelligence (AI), MRI can automatically analyze subtle changes in the images, not only improving diagnostic accuracy but also optimizing image processing, such as noise reduction and image enhancement. The integration of AI with MRI represents a significant advancement in medical imaging, making diagnoses more accurate and efficient, particularly in the early detection of bone diseases. The study by Deniz et al. proposed an automatic proximal femur segmentation method based on DCNN to enhance the clinical application of MRI in bone quality measurement and fracture risk assessment. The study used an MRI dataset from 86 subjects, and after CNN training and four-fold cross-validation, the segmentation results achieved a high Dice similarity coefficient of 0.95, with both precision and recall at 0.95 (Deniz et al., 2018). This suggests that the CNN method can effectively improve the accuracy of bone measurement, aiding in the clinical management of osteoporosis. Yabu et al. developed a CNN-based method for detecting fresh osteoporotic vertebral fractures (OVF), using 1,624 T1-weighted MR images from 814 patients for training and validation. By integrating VGG16, VGG19, DenseNet201, and ResNet50, the model achieved a ROC curve AUC of 0.949, with sensitivity, specificity, and accuracy of 88.1, 87.9, and 88.0%, respectively (Yabu et al., 2021). The study demonstrates the potential of CNN in accurately detecting fresh osteoporotic vertebral fractures, significantly aiding early diagnosis and intervention. Using multiple CNN architectures also enhances the model’s robustness and performance. Overall, MRI combined with AI holds potential in bone quality assessment and fracture risk prediction, but existing studies have small sample sizes, and the generalizability of the results has yet to be validated. Therefore, the clinical application of MRI in OP and fracture-related diseases still requires further validation through large-scale, multi-center clinical trials to confirm its effectiveness and feasibility.

## 4 Summary and outlook

AI technology plays a crucial role in the diagnosis of osteoporosis. Through ML, DL, and image processing, AI can handle vast amounts of medical data, enabling precise bone density evaluation and fracture risk prediction, thus assisting doctors in making informed decisions. Research shows that AI, combined with traditional imaging technologies such as X-rays, DXA, CT, and MRI, can significantly improve diagnostic accuracy, particularly in early screening and risk assessment, where it demonstrates tremendous potential. However, the practical clinical application of AI still faces several challenges that need to be addressed. First, the issue of AI model interpretability remains unresolved. Despite significant achievements of DL in medical image analysis, its “black box” nature limits the acceptance and trust of clinical practitioners (Dhar et al., 2023). Future research should focus on improving model transparency by using interpretability techniques to help doctors understand the rationale behind model decisions, thereby better integrating AI into the clinical decision-making process. Secondly, the lack of diversity in training data is a major bottleneck restricting the development of AI. Most existing studies are based on data from a single region, ethnicity, or age group, which may result in the model performing unevenly across different populations (Shams et al., 2025). More diverse and global training data are needed in the future to ensure that AI can be applied effectively across different groups. In addition, the lack of clinical validation also hinders the widespread adoption of AI technology. While laboratory data show excellent performance, the complexity of real-world clinical environments presents additional challenges for AI models (van de Sande et al., 2024). Prospective clinical trials must be conducted to validate the actual impact of AI models on patient treatment outcomes, ensuring their feasibility in real-world settings. In addition, the ethical issues and data security concerns of AI must be given high attention. Ensuring patient data privacy, avoiding biases in AI model decisions, and maintaining ethical standards in the use of AI are all important issues that cannot be overlooked in the future application of AI. Finally, as AI continues to advance in the diagnosis and treatment of osteoporosis, optimizing personalized treatment plans will be key to future development. By integrating multimodal data, AI can tailor personalized treatment plans for different patients, thus improving treatment outcomes and patients’ quality of life.
